# Epidemiological research on diabetic nephropathy at global, regional, and national levels from 1990 to 2021: an analysis derived from the global burden of disease 2021 study

**DOI:** 10.3389/fendo.2025.1647064

**Published:** 2025-08-27

**Authors:** Lu Zhang, Liangliang Jiang, Rong Xu, Xuemei Zhang, Boxun Zhang, Rensong Yue

**Affiliations:** Hospital of Chengdu University of Traditional Chinese Medicine, Chengdu, Sichuan, China

**Keywords:** diabetic nephropathy, global disease burden, epidemiology, GBD2021, forecasting

## Abstract

**Objective:**

A comprehensive assessment of the disease burden is essential for developing effective strategies to address diabetic nephropathy. This study investigates the long-term global trends and epidemiological characteristics of diabetic nephropathy.

**Methods:**

Data on diabetic nephropathy from the Global Burden of Disease (GBD) 2021 were utilized to evaluate morbidity, mortality, disability-adjusted life years (DALYs), and the impact of the Socio-Demographic Index (SDI). Global risk attribution was assessed, and the Bayesian Age–Period–Cohort (BAPC) model was applied to forecast the future burden of diabetic nephropathy.

**Results:**

In 2021, there were 107.6 million prevalent cases of diabetic nephropathy globally (95% UI: 99.2–116.0), with an age-standardized prevalence rate of 1,259.6 per 100,000 population (95% UI: 1,162.0–1,359.9), representing a 5.1% decline since 1990. Global deaths attributed to diabetic nephropathy in 2021 reached 477.3 thousand (95% UI: 401.5–566.0), with an age-standardized mortality rate of 5.7 per 100,000 (95% UI: 4.8–6.8), reflecting a 37.8% increase since 1990. The number of DALYs attributable to diabetic nephropathy was 11,278.9 thousand (95% UI: 9,682.8–13,103.9), with an age-standardized DALY rate of 131.1 per 100,000 (95% UI: 112.8–152.5), indicating a 24% rise since 1990.

**Conclusions:**

Over the past three decades, the global age-standardized prevalence of diabetic nephropathy has declined, while age-standardized mortality and DALY rates have increased. Significant disparities exist in prevalence, incidence, and DALY rates across regions and countries. The SDI exerts a notable influence on diabetic nephropathy prevalence, underscoring the importance of sustained and enhanced management of risk factors to prevent and treat this condition. Diabetic nephropathy remains a critical global health challenge moving forward.

## 1 Introduction

Diabetic nephropathy (DN), also referred to as diabetic kidney disease (DKD), is a major microvascular complication of diabetes mellitus and a leading cause of chronic kidney disease and end-stage renal disease (1, 2). Approximately 40% of individuals with diabetes develop DN (3). The pathogenesis of DN is complex, involving metabolic disturbances driven by chronic inflammation, oxidative stress, and persistent hyperglycemia (4, 5). Clinically, DN is characterized by a progressive decline in glomerular filtration rate, thickening of the glomerular basement membrane, worsening proteinuria, glomerular hypertrophy, podocyte loss, and hyperplasia of associated membranes (6–8). According to the International Diabetes Federation, more than 460 million people worldwide are currently living with diabetes, and DN is projected to become a major global public health challenge (9). Patients with DN often require lifelong dialysis or kidney transplantation, resulting in substantial socioeconomic burden (10).

The Global Burden of Disease (GBD) study provides comprehensive epidemiological data that offer critical insights for public health policymaking (11). The GBD database contains extensive information on prevalence, mortality, and disability-adjusted life years (DALYs) from 1990 to 2021, serving as an essential resource for assessing global trends in DN. This study aimed to leverage GBD data to analyze epidemiological patterns of DN over this period and to project future trends in its burden, thereby providing evidence to inform public health strategies and clinical interventions.

## 2 Materials and methods

### 2.1 Sources of data

The GBD 2021 database encompasses the most recent global and regional epidemiological data on 371 diseases and injuries, along with 88 associated risk factors (12). These data are publicly accessible through the Global Health Data Exchange (GHDx) query tool (https://vizhub.healthdata.org/gbd-results/). For this study, we extracted global data on DN, including information on age, incidence, mortality, and DALYs.

### 2.2 Statistical analysis and visualization

This study employed prevalence, incidence, mortality, disability-adjusted life years (DALYs), and estimated annual percentage change (EAPC) to assess epidemiological trends in DN. Global burden data for DN from 1990 to 2021 were compiled in Excel 2024 and primarily analyzed using the Bayesian Age–Period–Cohort (BAPC) prediction model. This model applies Bayesian inference to integrate age, period, and cohort effects, enabling the projection of long-term trends. The strength of the BAPC model lies in its capacity to address data sparsity and heterogeneity, thereby producing more robust predictive estimates. Model construction and inference were performed using the BAPC package in RStudio, with parameter estimation conducted via the Markov Chain Monte Carlo (MCMC) method. The influence of the socio-demographic index (SDI) on DN was also evaluated. All statistical analyses and data visualizations were performed using R software, version 4.4.1.

## 3 Results

### 3.1 International scale

In 2021, the global number of prevalent cases of DN was estimated at 107.6 million (95% UI: 99.2–116.0), corresponding to an age-standardized prevalence rate of 1,259.6 per 100,000 population (95% UI: 1,162.0–1,359.9), representing a 5.1% decrease since 1990. In the same year, global deaths attributable to DN reached 477.3 thousand (95% UI: 401.5–566.0), with an age-standardized mortality rate of 5.7 per 100,000 population (95% UI: 4.8–6.8), marking a 37.8% increase since 1990. The global DALYs associated with DN in 2021 totaled 11,278.9 thousand (95% UI: 9,682.8–13,103.9), with an age-standardized rate of 131.1 per 100,000 population (95% UI: 112.8–152.5), reflecting a 24.0% increase since 1990 (**Table 1**).

**Table 1:** The prevalence, mortality, and DALYs of DN in 2021, as well as the percentage change in age-standardized rates (ASR) per 100,000 people in global disease burden regions from 1990 to 2021.

	Location	Prevalence (95% UI)	ASRs per 100000 (95% UI)	Percentage change in ASRs from 1990 to 2021	Deaths (95% UI)	ASRs per 100000 (95% UI)	Percentage change in ASRs from 1990 to 2021	DALYs (95% UI)	ASRs per 100000 (95% UI)	Percentage change in ASRs from1990 to 2021
		No, in millions (95% UI)			No, in thousands (95% UI)			No, in thousands (95% UI)		
1	Global	107.6 (99.2,116)	1259.6 (1162,1359.9)	-5.1 (-7.5,-3)	477.3 (401.5,566)	5.7 (4.8,6.8)	37.8 (19.2,49.6)	11278.9 (9682.8,13103.9)	131.1 (112.8,152.5)	24 (9.3,33)
2	High-income Asia Pacific	4.6 (4.2,4.9)	1275.4 (1168.2,1377.4)	-11.8 (-13.6,-10)	23.6 (17.3,30.4)	3.6 (2.8,4.5)	-18.3 (-25.2,-13)	394.5 (318.7,468.5)	75.2 (62.2,87.2)	-18.9 (-23.2,-15.1)
3	High-income North America	6.2 (5.8,6.7)	1056.5 (979.6,1135.3)	4.9 (3.5,6.4)	57.2 (49.3,64.5)	8.1 (7,9.1)	259.9 (210.8,316.3)	1153.7 (1037.9,1260.9)	174 (157,190.9)	168.4 (142.9,199.9)
4	Western Europe	5.9 (5.4,6.3)	737.4 (683,790.1)	-10.7 (-14.4,-8)	22.8 (16.6,30.9)	1.8 (1.4,2.4)	30.6 (16.1,44.3)	428.6 (343.3,532.2)	40.9 (32.9,49.7)	5.6 (-1.4,12.7)
5	Australasia	0.4 (0.3,0.4)	768.6 (695,843.2)	-7.1 (-12.3,-3.6)	0.5 (0.4,0.7)	0.8 (0.6,1.1)	62.5 (42.3,85.2)	13 (10.1,16.2)	23.6 (18.3,29.5)	29 (15.6,44.9)
6	Andean Latin America	0.6 (0.5,0.7)	957.2 (862.9,1056.2)	-2.7 (-5.8,0.3)	8.4 (6.5,10.8)	14.9 (11.5,19.2)	42.8 (15.1,76.5)	165.5 (127.5,211.1)	286.1 (220.7,365.1)	36.4 (11.6,68.7)
7	Tropical Latin America	3 (2.7,3.2)	1145.8 (1053.3,1238.7)	-9.5 (-11.9,-7.5)	17.9 (15,21.2)	7.2 (6,8.6)	16.4 (10.2,21.5)	397.8 (335.7,457.7)	155.9 (131.5,178.9)	8.6 (3.6,13)
8	Central Latin America	3.4 (3.1,3.7)	1327.6 (1225,1427.2)	-5.7 (-8.3,-3.3)	24.4 (19.3,30.5)	10.1 (8,12.5)	49.9 (37,64.6)	582.7 (462.7,718.5)	232.6 (185.5,287.1)	56.6 (41.8,72)
9	Southern Latin America	0.8 (0.7,0.8)	911.8 (819.8,1021.1)	3 (-1,6.6)	4.6 (3.6,5.8)	5 (3.9,6.3)	-2.8 (-10.5,4.9)	88.5 (71.1,108.3)	99.6 (80.2,121.6)	-8.3 (-13.9,-2.3)
10	Caribbean	0.6 (0.5,0.6)	1069.9 (978.5,1169.7)	-4.1 (-6.9,-1.4)	6.2 (5.2,7.5)	11.4 (9.5,13.7)	43 (24,61.8)	131.4 (108.8,159.2)	242.9 (200.8,294.5)	40.1 (21.4,59)
11	Central Europe	1.7 (1.6,1.8)	855.7 (792.6,922.2)	-8.8 (-12.5,-6.3)	2.8 (2.1,3.5)	1.1 (0.9,1.4)	5.4 (-4.8,16.6)	76 (62.1,92.5)	33.5 (27.3,40.6)	-2 (-9,5.6)
12	Eastern Europe	4.3 (4,4.7)	1390.3 (1277.4,1520.4)	-9.6 (-13.9,-6.5)	2.8 (2.2,3.6)	0.8 (0.6,1)	142.7 (115.2,168.5)	95.8 (76.3,118.8)	26.7 (21.4,33)	25.7 (13.4,40)
13	Central Asia	1.3 (1.2,1.4)	1494 (1381.4,1617.5)	-4.5 (-7.8,-1.7)	1.3 (1,1.7)	1.8 (1.3,2.3)	177.9 (122.1,241.4)	54.9 (43.8,67.8)	67.5 (54.1,82.9)	52.9 (35.6,72.5)
14	North Africa and Middle East	8 (7.3,8.8)	1505.9 (1369,1642.9)	-4 (-6.4,-1.7)	31 (23.7,39.8)	8.2 (6.3,10.5)	28.9 (-18.2,60.8)	739.7 (577.4,932.7)	170.2 (133.9,214.5)	22.3 (-18.8,49.4)
15	South Asia	25.5 (23.2,28)	1547.3 (1418.4,1687.6)	-9.7 (-12,-7.5)	70.3 (54.7,89.9)	5.3 (4.1,6.6)	28.5 (-0.7,55.5)	1976.8 (1609.6,2453.2)	134.4 (110.5,166)	22.1 (-0.6,43.7)
16	Southeast Asia	12.3 (11.3,13.4)	1739.3 (1595.7,1883.9)	-3 (-5.5,-0.7)	59 (48.6,70.6)	10.4 (8.6,12.5)	31.4 (5.6,52.1)	1535.4 (1287.5,1818.9)	237.7 (199.8,276.1)	23.5 (2.9,40.5)
17	East Asia	21.7 (19.9,23.4)	1054.1 (972.3,1140.1)	-13.1 (-15.7,-10.9)	115.1 (91.6,141.5)	5.8 (4.7,7.2)	-16.6 (-35.7,1.5)	2697.3 (2197.7,3240.4)	125.6 (103.4,149.7)	-20.9 (-35.6,-5.9)
18	Oceania	0.1 (0.1,0.1)	1337.2 (1193.9,1478.5)	-4.3 (-7.6,-1.3)	0.8 (0.6,1)	13.6 (11.2,17.3)	27.3 (-10.2,80.5)	22.6 (18.3,27.9)	309.8 (257.3,383.9)	23.8 (-10.4,71.1)
19	Western Sub-Saharan Africa	3.2 (3,3.5)	1276.3 (1183.6,1373.8)	-3.9 (-6,-2)	8.1 (6.1,10.9)	5.5 (4.1,7.5)	11.4 (-7.2,29.3)	222.8 (175,283.1)	124.3 (97.5,159.9)	7.6 (-6.5,22.6)
20	Eastern Sub-Saharan Africa	2.2 (2,2.4)	942.8 (861.6,1032.9)	-1.1 (-4.1,1.1)	15.1 (12.1,18.9)	12 (9.7,15)	-4.9 (-18.5,9.5)	344.1 (277.4,431.6)	230.4 (187.1,284.8)	-13.5 (-23.9,-1.4)
21	Central Sub-Saharan Africa	1 (0.9,1.1)	1377.5 (1262.9,1506.7)	-6.9 (-9.8,-4.1)	3.6 (2.5,5)	9 (6.1,12.9)	4.8 (-22.4,36.5)	97.6 (69.6,134.1)	196.1 (139.9,268.7)	2.4 (-20.6,31.3)
22	Southern Sub-Saharan Africa	0.9 (0.8,1)	1362.7 (1254.9,1471)	-3.9 (-6.4,-1.6)	2.1 (1.6,2.8)	4.4 (3.3,5.8)	49.3 (10.3,73.8)	60.3 (47.5,78.2)	108.9 (85.5,140)	36.9 (11.3,55)

### 3.2 Regional tier

In 2021, the highest age-standardized prevalence rates (per 100,000 population) of DN were observed in Southeast Asia (1,739.3), South Asia (1,547.3), North Africa and the Middle East (1,505.9), and Central Asia (1,494.0). In contrast, Western Europe (737.4), Australasia (768.6), and Central Europe (855.7) reported comparatively lower prevalence rates (**Table 1**). During the same year, Andean Latin America (14.9), Oceania (13.6), and Eastern Sub-Saharan Africa (12.0) recorded the highest age-standardized mortality rates (per 100,000), whereas Eastern Europe (0.8), Australasia (0.8), and Central Europe (1.1) had the lowest (**Table 1**). Similarly, Oceania (309.8), Andean Latin America (286.1), and the Caribbean (242.9) exhibited the highest age-standardized DALY rates (per 100,000), while Central Europe (33.5), Western Europe (40.9), and Central Asia (67.5) recorded the lowest (**Table 1**).

From 1990 to 2021, the increase in age-standardized prevalence of DN was most pronounced in High-income North America (4.9%) and Southern Latin America (3.0%), whereas the largest declines were observed in East Asia (−13.1%) and High-income Asia Pacific (−11.8%) (**Table 1**). During the same period, age-standardized mortality rates rose substantially in High-income North America (259.9%), Central Asia (177.9%), and Eastern Europe (142.7%), while notable decreases were recorded in East Asia (−16.6%), High-income Asia Pacific (−18.3%), and Eastern Asia (−16.3%). Age-standardized DALY rates increased markedly in High-income North America (168.4%), Central Latin America (56.6%), and Central Asia (52.9%), but declined in East Asia (−20.9%), High-income Asia Pacific (−18.9%), and Eastern Sub-Saharan Africa (−13.5%) (**Table 1**).

### 3.3 National level

**Figure 1:**
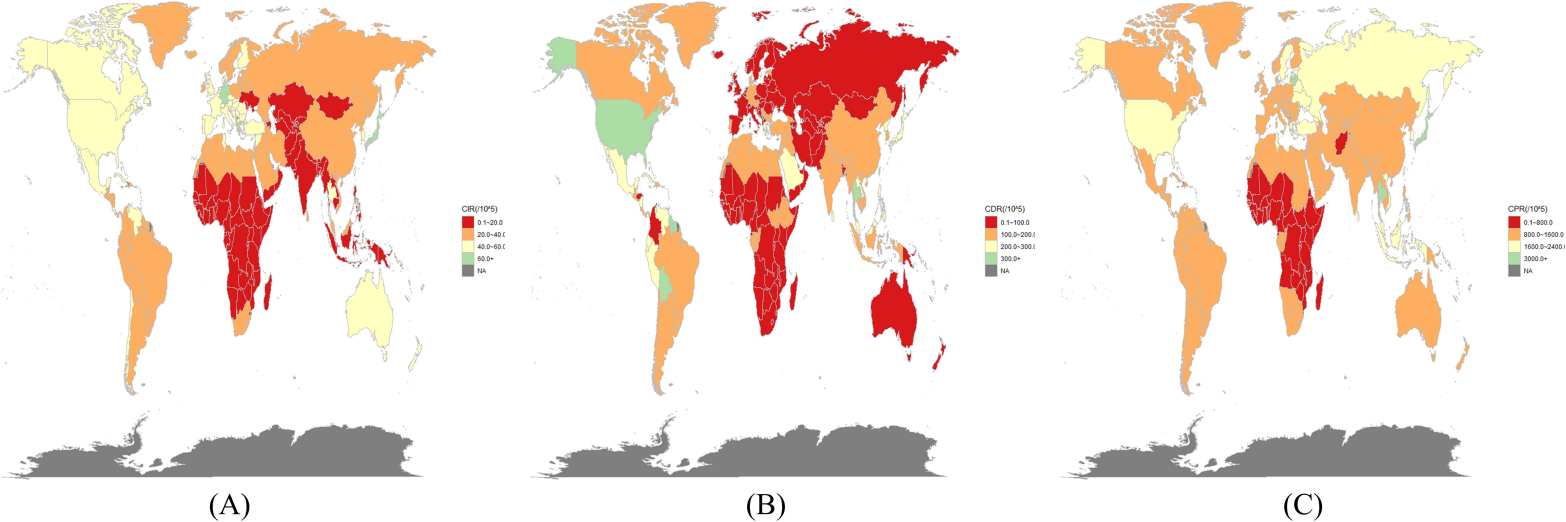
In 2021, the burden of diabetic nephropathy was estimated in 204 countries/territories. **(A)** incidence, **(B)** DALY rates and **(C)** prevalence.

In 2021, countries with the highest incidence rates included Japan (82.96; 95% UI: 74.83–90.76), Puerto Rico (68.94; 95% UI: 60.32–78.53), and Bermuda (67.59; 95% UI: 58.21–76.43) (**Figure 1A**). The highest age-standardized DALY rates (per 100,000 population) were recorded in Mauritius (1,071.04; 95% UI: 918.15–1,229.15), American Samoa (978.03; 95% UI: 748.02–1,221.06), and Niue (950.07; 95% UI: 567.49–1,434.78) (**Figure 1B**).The highest age-standardized prevalence rates (per 100,000 population) of DN were observed in Japan (2,764.19; 95% UI: 2,539.65–2,976.44), Thailand (2,716.64; 95% UI: 2,417.13–3,019.55), Mauritius (2,569.61; 95% UI: 2,256.73–2,900.06), and the Republic of Moldova (2,533.64; 95% UI: 2,262.28–2,826.01) (**Figure 1C**).

### 3.4 Demographic patterns of age and gender

**Figure 2:**
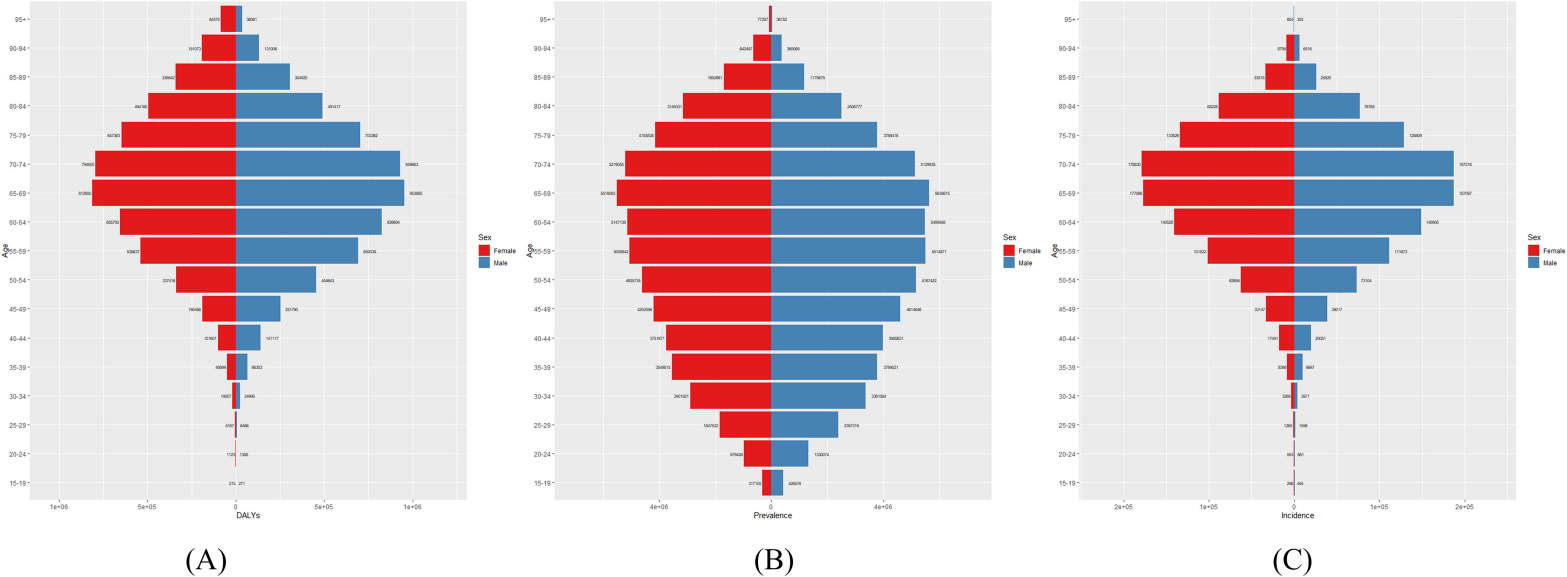
Global **(A)** Number of DALYs, **(B)** Number of Prevalences, and **(C)** Number of Incidences of diabetic nephropathy in 2021.

**Figure 3:**
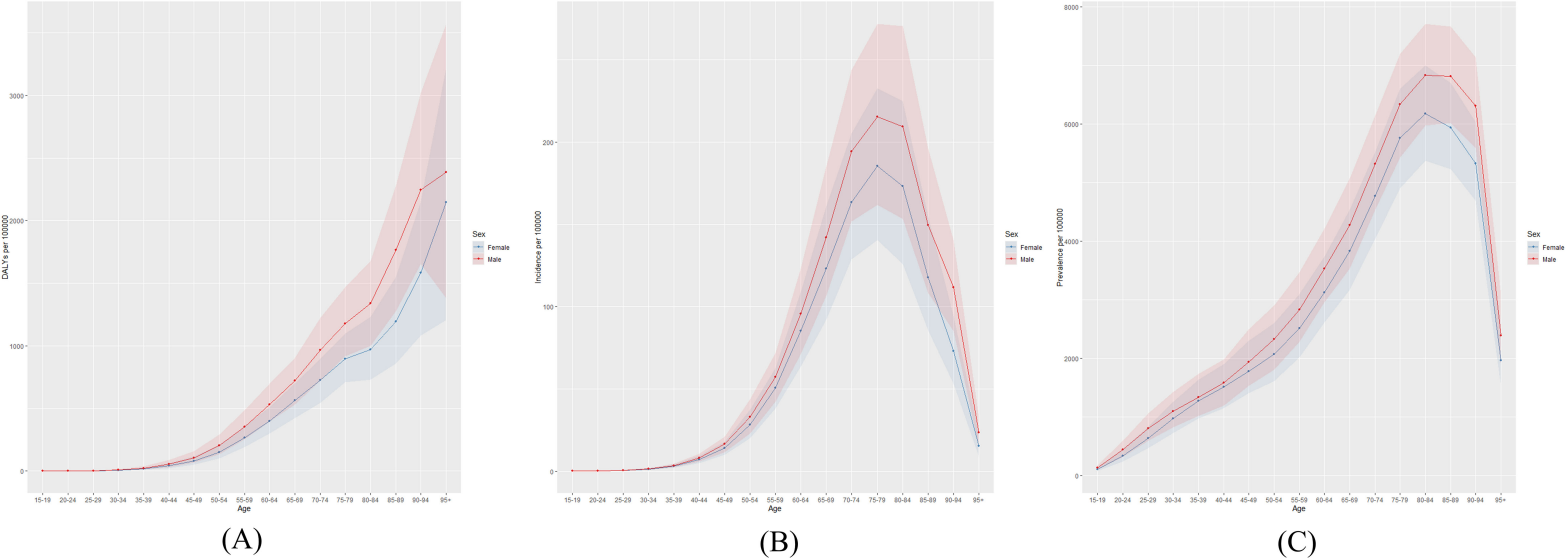
Global **(A)** age-standardized DALY rate, **(B)** age-standardized incidence rate, and **(C)** age-standardized prevalence rate of diabetic nephropathy in 2021.

**Figure 2** presents the global number of prevalent cases, incident cases, and DALYs for DN by age group in 2021. The highest number of prevalent cases was observed in individuals aged 65–69 years, while the highest number of incident cases occurred in the 70–74-year age group. **Figure 3** depicts the global age-specific prevalence, incidence, and DALY rates of DN in 2021. Males demonstrated a higher susceptibility to DN than females. The disease burden increases progressively after the age of 45, reaching its peak between 75 and 79 years.

### 3.5 Correlation with SDI

**Figure 4:**
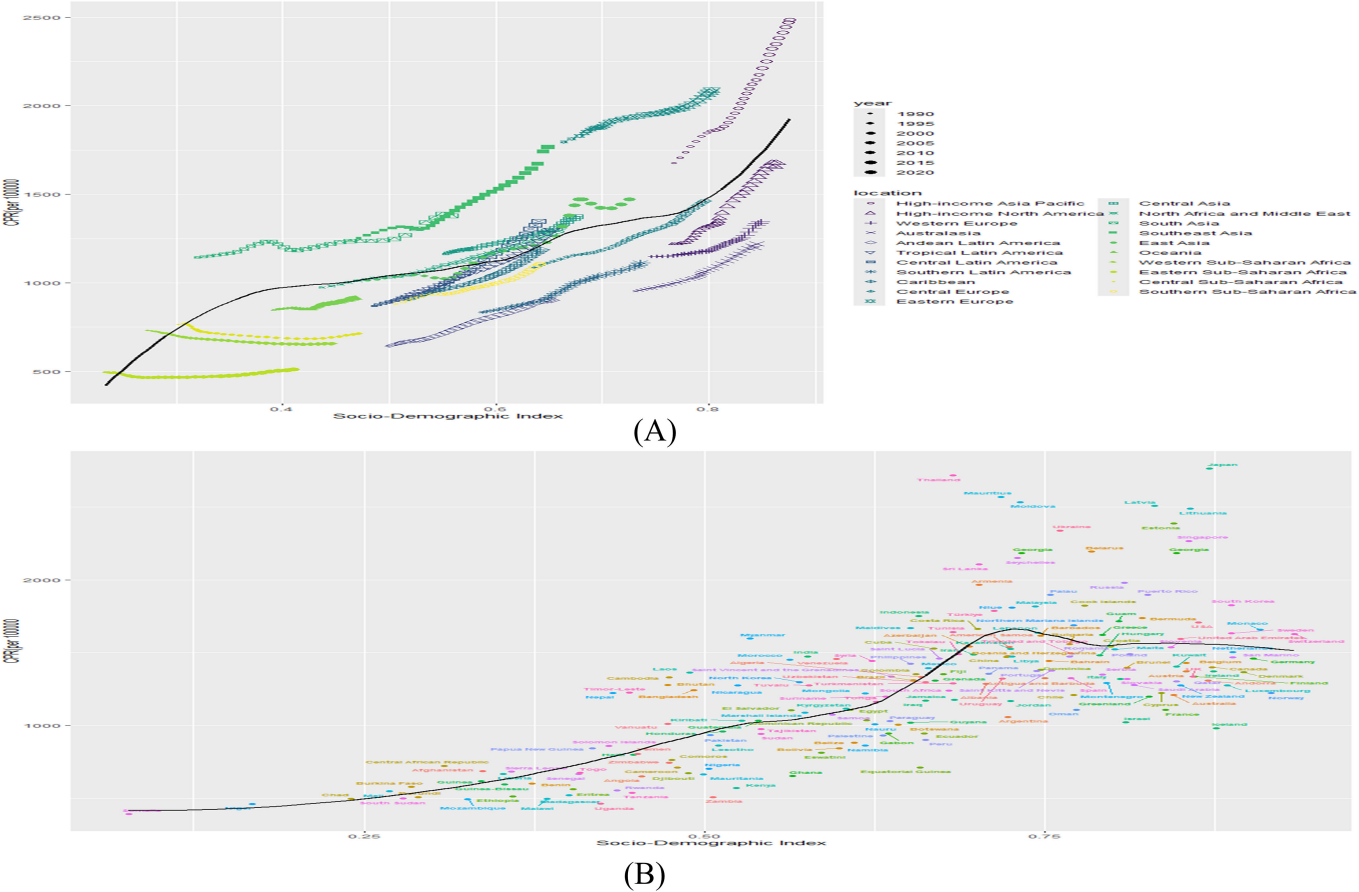
**(A)** Association of prevalence with SDI by region, 1990-2021. **(B)** Association of prevalence with SDI by country in 2021. (B&L represents expected prevalence based on SDI only).

At the regional level, an S-shaped association was observed between the SDI and the prevalence of DN from 1990 to 2021, with prevalence increasing sharply as the SDI rose. South Asia, Southeast Asia, Eastern Europe, and High-income Asia Pacific exhibited prevalence rates exceeding those expected based on SDI (**Figure 4A**). At the national level, a V-shaped relationship was identified between DN prevalence and SDI, with rates surpassing expectations in Thailand, Mauritania, Latvia, Japan, and Lithuania (**Figure 4B**).

### 3.6 Risk factors

**Figure 5:**
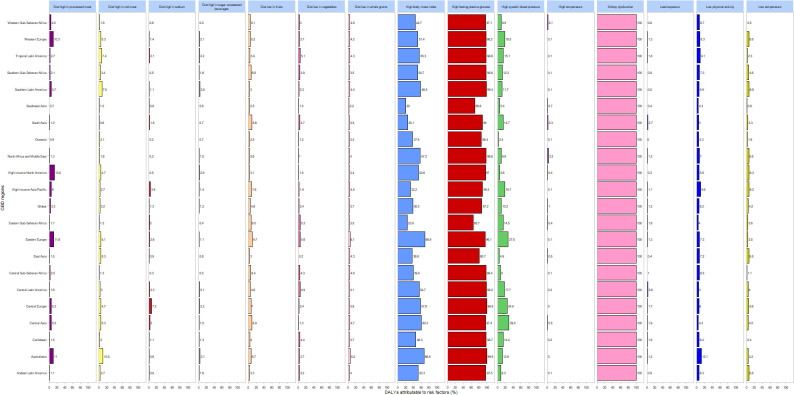
DALYs for diabetic nephropathy and risk factor shares in 21 regions in 2021.

**Figure 5** depicts the proportions of risk factors contributing to DALYs attributable to DN globally and across 21 regions in 2021. The leading risk factors include renal impairment, elevated fasting plasma glucose, and increased body mass index. Diets high in processed meat predominantly affect Western Europe and other high-income regions, while diets rich in red meat mainly impact Australasia. Elevated systolic blood pressure is a primary risk factor in Central Asia, Central Europe, and Eastern Europe.

### 3.7 Projections for the future

**Figure 6:**
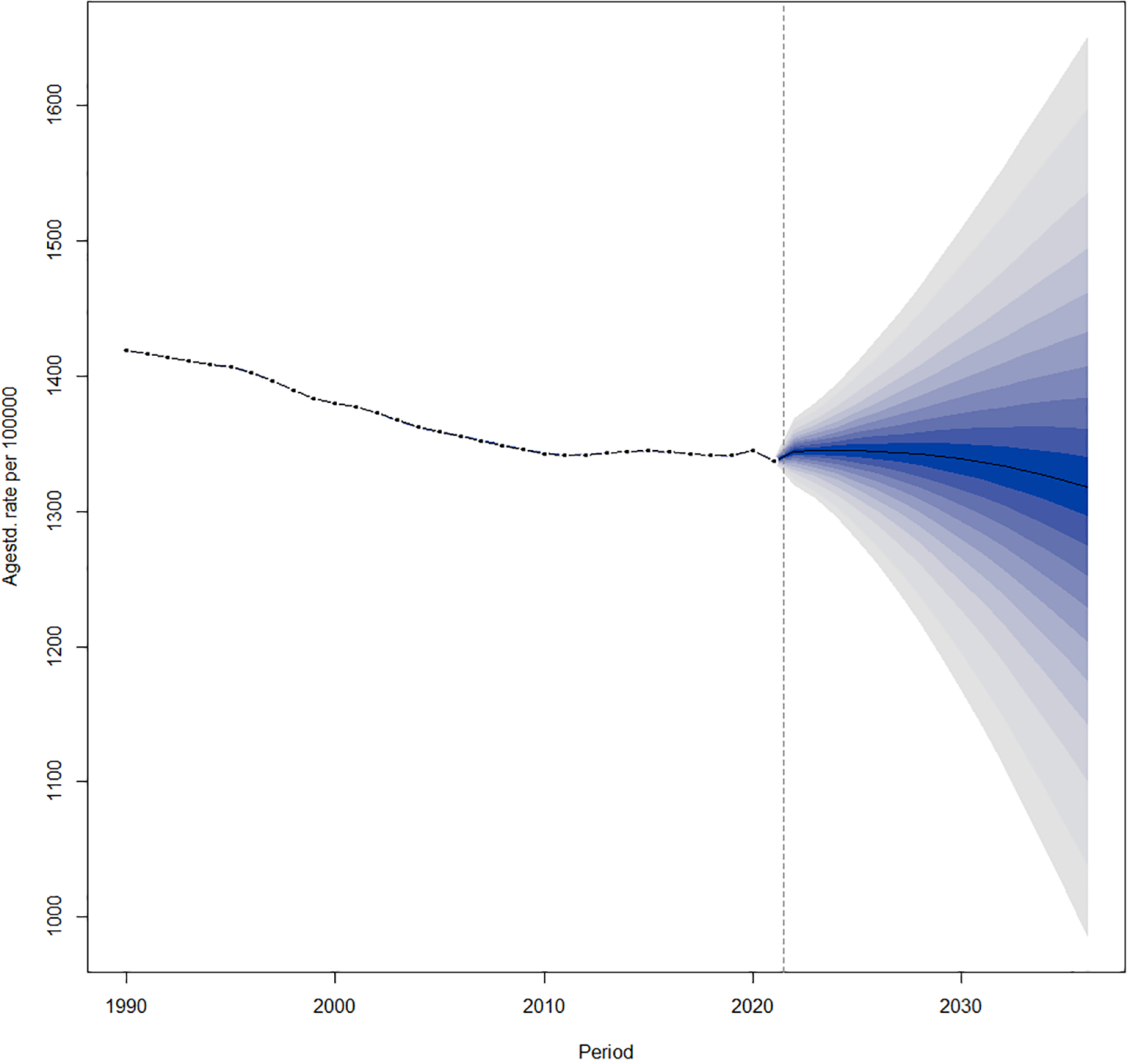
Temporal trends in global age-standardized prevalence from 1990 to 2036.

The age-standardized prevalence of DN is declining globally from 2022 to 2036 (**Figure 6**).

## 4 Discussion

### 4.1 Principal discoveries

This study utilizes data from the Global Burden of Disease Study 2021 to provide comprehensive estimates of the prevalence, mortality, DALYs, and age-standardized rates of DN from 1990 to 2021. In 2021, an estimated 107.6 million cases, 477,300 deaths, and 11.28 million DALYs were attributed to DN worldwide. The burden of DN was analyzed at global, regional, and national levels using contemporary epidemiological approaches and risk factor assessment. These findings offer valuable insights into the impact of varying SDI levels on DN, thereby supporting policymakers in developing targeted prevention and management strategies.

### 4.2 Comparison with alternative research

A 2021 study reported a 0.81% increase in the age-standardized DALY rate for DN (13). From 1990 to 2021, the global age-standardized prevalence of DN decreased by 5.1%, whereas the age-standardized mortality rate increased by 37.8%. Our study provides additional critical insights. Although a previous analysis did not assess risk factors or stratify by age and sex, its findings on mortality, prevalence, and DALYs generally correspond with ours (14). This underscores that our study offers the most comprehensive and precise data currently available.

From 1990 to 2021, global mortality and DALY rates for DN steadily increased, highlighting its emergence as a significant public health concern. Despite advances, the global management of DN faces ongoing challenges. Key strategies to mitigate its impact include stringent glycemic control (15, 16), lipid management (17), blood pressure regulation (18, 19), dietary modifications (20), and careful use of nephrotoxic medications (21, 22). Recent studies into the pathophysiology of DN have elucidated mechanisms such as renal structural alterations (23), glomerular hyperfiltration (24), inflammation (25), lipotoxicity (26), organelle dysfunction (27), and vascular abnormalities (28). Further comprehensive research is essential to improve therapeutic approaches and patient outcomes in DN.

At the regional level, Southeast Asia exhibits the highest prevalence of DN. This predominantly developing region is marked by uneven economic development and accounts for approximately 1% of global health expenditure (29). Southeast Asia currently faces significant societal challenges, including rising obesity rates, rapid population aging, and urbanization. Sedentary lifestyles and dietary patterns prevalent in the region further contribute to the increasing burden of DN (30). In contrast, Western Europe, characterized by the lowest prevalence,ranks among the wealthiest regions globally and benefits from an advanced healthcare system with well-established renal care protocols that support effective prevention and management of DN (31). The density of healthcare professionals in Western Europe considerably exceeds the global average (32), thereby improving the quality of DN care. Additionally, Western European governments provide substantial financial support for renal disease-related healthcare costs, which are markedly higher than the global average (33).

In 2021, Japan, characterized by an aging population, exhibited the highest prevalence of DN. Individuals of Japanese descent have been shown to possess a reduced capacity for insulin secretion (34). Coupled with an aging demographic and increasing adoption of Westernized lifestyles and dietary habits, this has contributed to the rising prevalence of DN. At least three genetic variants have been identified as contributing factors to DN within the Japanese population (35). As a developed nation equipped with advanced medical technologies, Japan’s high prevalence figures likely reflect robust early screening and diagnostic practices. The elevated prevalence of DN in Japan thus reflects the interplay of physiological, genetic, and environmental factors unique to this population.

Research indicates that diets high in processed meat, red meat, salt, and sugar-sweetened beverages are significant risk factors for the development of diabetic kidney disease. Addressing these modifiable risk factors is essential for the prevention and management of DN. Diet plays a critical role in the pathogenesis of DN (36, 37). Economically advanced regions such as Western Europe and High-income North America typically consume protein-rich diets, which may contribute to disease progression. Limiting protein intake has been shown to support the prevention and management of DN (36, 38). Conversely, diets rich in fruits and vegetables provide essential vitamins that may confer renal protection (39). Elevated fasting plasma glucose promotes inflammation, glomerulosclerosis, and renal fibrosis, thereby accelerating DN progression (40). Future strategies aimed at optimizing protein intake and supplementing with vitamin-rich foods may help alleviate the global burden of DN.

This study also identified a significant association between the burden of DN and the SDI. Our findings demonstrate that the prevalence of DN increases with rising SDI at the regional level, with a higher burden observed in economically developed regions. At the national level, DN prevalence was elevated in countries with medium to high SDI, where advanced medical technologies facilitate accurate diagnosis. Conversely, countries and regions with low SDI face technical limitations and challenges in the detection and diagnosis of DN.

This study’s strength lies in its systematic and comprehensive evaluation of the epidemiology of DN at global, regional, and national levels from 1990 to 2021. It enables assessment of the worldwide impact of DN by comparing regions and countries with varying healthcare resources. There is a critical need to enhance systematic, effective, and routine screening and renal function monitoring for DN (41). Regions and countries with a high prevalence of DN should allocate additional healthcare resources and implement tailored policies to improve prevention and management efforts. However, this study has several limitations. The data used in this study are not original data, but rather GBD data on DN caused by type 2 diabetes. The data were derived from the GBD database, which relies on varying data collection capacities across countries and regions. Inconsistencies in data quality and the absence of source data in some settings may have affected the accuracy of our analysis.

## 5 Conclusion

This study examines the current status and trends in the global epidemiology of DN over the past three decades. Despite a decline in age-standardized prevalence, both age-standardized mortality and DALY rates have increased. Substantial variation exists in prevalence, incidence, and DALY rates across regions and countries. The SDI exerts a significant influence on DN prevalence, and sustained improvements in risk factor management are essential to mitigate its impact. DN is expected to remain a major global health challenge in the future.

## Data Availability

Publicly available datasets were analyzed in this study. This data can be found here: The data for this study are available in the GBD database (https://vizhub.healthdata.org/gbd-results/).
